# Pathogenomes and variations in Shiga toxin production among geographically distinct clones of *Escherichia coli* O113:H21

**DOI:** 10.1099/mgen.0.000796

**Published:** 2022-04-08

**Authors:** Anna Allué-Guardia, Sara S. K. Koenig, Ricardo A. Martinez, Armando L. Rodriguez, Joseph M. Bosilevac, Peter Feng†, Mark Eppinger

**Affiliations:** ^1^​ Department of Molecular Microbiology and Immunology, University of Texas at San Antonio, San Antonio, TX, USA; ^2^​ South Texas Center for Emerging Infectious Diseases (STCEID), San Antonio, TX, USA; ^3^​ University of Texas at San Antonio, Research Computing Support Group, San Antonio, TX, USA; ^4^​ U.S. Department of Agriculture (USDA), Agricultural Research Service (ARS), Roman L. Hruska U.S. Meat Animal Research Center, Clay Center, NE, USA; ^5^​ U.S. Food and Drug Administration (FDA), College Park, MD, USA

**Keywords:** Shiga toxin (Stx) producing *Escherichia coli *(STEC), O113:H21 serotype, long-read technology (LRT), Whole Genome Sequencing and Typing (WGST), Multilocus Sequence Type (MLST), phylogenomics, genomics epidemiology

## Abstract

Infections with globally disseminated Shiga toxin-producing *Escherichia coli* (STEC) of the O113:H21 serotype can progress to severe clinical complications, such as hemolytic uremic syndrome (HUS). Two phylogeographically distinct clonal complexes have been established by multi locus sequence typing (MLST). Infections with ST-820 isolates circulating exclusively in Australia have caused severe human disease, such as HUS. Conversely, ST-223 isolates prevalent in the US and outside Australia seem to rarely cause severe human disease but are frequent contaminants. Following a genomic epidemiology approach, we wanted to gain insights into the underlying cause for this disparity. We examined the plasticity in the genome make-up and Shiga toxin production in a collection of 20 ST-820 and ST-223 strains isolated from produce, the bovine reservoir, and clinical cases. STEC are notorious for assembly into fragmented draft sequences when using short-read sequencing technologies due to the extensive and partly homologous phage complement. The application of long-read technology (LRT) sequencing yielded closed reference chromosomes and plasmids for two representative ST-820 and ST-223 strains. The established high-resolution framework, based on whole genome alignments, single nucleotide polymorphism (SNP)-typing and MLST, includes the chromosomes and plasmids of other publicly available O113:H21 sequences and allowed us to refine the phylogeographical boundaries of ST-820 and ST-223 complex isolates and to further identify a historic non-shigatoxigenic strain from Mexico as a quasi-intermediate. Plasmid comparison revealed strong correlations between the strains’ featured pO113 plasmid genotypes and chromosomally inferred ST, which suggests coevolution of the chromosome and virulence plasmids. Our pathogenicity assessment revealed statistically significant differences in the Stx_2a_-production capabilities of ST-820 as compared to ST-223 strains under RecA-induced Stx phage mobilization, a condition that mimics Stx-phage induction. These observations suggest that ST-820 strains may confer an increased pathogenic potential in line with the strain-associated epidemiological metadata. Still, some of the tested ST-223 cultures sourced from contaminated produce or the bovine reservoir also produced Stx at levels comparable to those of ST-820 isolates, which calls for awareness and for continued surveillance of this lineage.

## Data Summary

The authors confirm all supporting data, code and protocols have been provided within the article or through supplementary data files.

**Impact Statement**

Shiga toxin-producing *Escherichia coli* (STEC) of serotype O113:H21 are a globally disseminated lineage, which can be partitioned into two major phylogeographical complexes: Sequence Type (ST)−820 strains reside in Australia and have been linked to severe outbreaks of human disease, while ST-223 strains are found outside Australia and are common adulterants, yet with unknown association to human disease. To discern potential differences in the pathogenome make-up and conferred pathogenicity associated with these clonal complexes, we assembled a representative collection of twenty O113:H21 strains. Informed by the sequenced genomes along with recorded Stx-production pathotypes, we were able to refine the phylogenomic and virulence boundaries associated with these two complexes. The established high-resolution framework (based on whole genome alignment, SNP-typing and MLST) allowed us to refine the phylogenomic boundaries between the two complexes and identify a historic non-shigatoxigenic O113:H21 strain from Mexico as quasi-intermediate. Our pathogenicity assessment supports the notion of an increased pathogenic potential of the HUS-associated ST-820 strains consistent with the known strain-associated epidemiological metadata. Insights into the genomic and phenotypic plasticity of STEC on a lineage- and genome-wide scale are foundational to improve and inform risk assessment, biosurveillance, and prevention strategies for STEC.

## Introduction

Shiga toxin (Stx)-producing *Escherichia coli* are notorious for producing a phage-borne toxin [1–5] that is specifically toxigenic towards renal endothelial cells [6–13]. Infections can progress to life-threatening complications, such as hemolytic uremic syndrome (HUS) [14]. Pathogenicity in humans is inexorably linked to the Stx litres produced by individual STEC strains [15, 16]. Hypervirulent clones, as manifested by increased Stx_2a_-litres [17–24], have been associated with STEC subpopulations through phylogenetic, epidemiological, and phenotypic linkage [4, 5, 11, 19, 20, 25–49]. The specific factors responsible for elevated Stx-production in hypervirulent STEC strains are unknown but presumably modulated by a number of biotic and abiotic triggers [6, 8, 50–54]. Globally disseminated STEC of serotype O113:H21 were first associated with HUS cases in 1983 [14]. The most potent cytopathic toxin subtype, Stx_2a_, is commonly found in the O113:H21 serotype [55, 56] that lacks the locus of enterocyte effacement (LEE) [57, 58]. Multilocus sequence typing (MLST) has identified two major phylogeographical complexes, where ST-223 strains are found around the world, while ST-820 strains are restricted to Australia [59, 60]. In particular, infections with Australian ST-820 strains have been associated with severe clinical complications [61–63], while ST-223 isolates from the US [55, 64] and elsewhere [65–67] have been rarely associated with severe human disease, even though they are frequent contaminants of produce and cattle [68–70] and can possess virulence traits similar to the clinical O113:H21 strains [59, 60]. There is a dearth of knowledge of the intrinsic genomic make-up and Stx-production associated traits of ST-223 and ST-820 complex isolates that might contribute to the disparity in human disease manifestation. To investigate the plasticity and to discern potential differences in the pathogenome and Stx-production traits, we assembled a collection of O113:H21 strains of global origin including strains from Australia, Asia, Europe and North and South America from clinical cases, cattle and contaminated produce. Following a genomic epidemiology approach, we sequenced a total of 20 O113:H21 strains and analysed their Stx virulence phenotypes alongside 15 other published genomes. Through comprehensive genotypic and phenotypic analyses, we determined the genome make-up and virulence traits associated with Stx-production in these representative ST-223 and ST-820 complex isolates and established a high-resolution phylogenomic framework. Integrating the virulence information to the genome plasticity within this lineage is foundational for improved risk assessment, biosurveillance, and the development of prevention strategies for STEC [71, 72].

## Methods

### Bacterial strains analysed in this study

A collection of O113:H21 cultures and genomes, representing the two major complexes ST-223 (#12) and ST-820 (#8), isolated from the bovine and environmental reservoir, produce, and clinical cases were sequenced and analysed in this study. Strain-associated metadata can be found in Table S1 (available in the online version of this article). The sequenced strains include eight Australian strains isolated from either HUS, thrombotic thrombocytopenic purpura (TTP), or dysentery patients, and nine strains from the US recovered from ground beef or spinach [68]. We further included Canadian strains CL-3 (AGTH01000000.1) and TW01391, the latter sequenced in this study, which are clones from different culture repositories; strain TW02918, isolated from a diarrhoea patient in Thailand, and strain 55 isolated from Uruguayan beef [59, 60, 73]. To support lineage-scale analyses we examined 15 additional O113:H21 genomes retrieved from NCBI GenBank/SRA: eight water-, cattle-, and swine strains from a major produce region in California [55, 64, 74], five US strains from four clinical cases and one from cattle faeces, and a single isolate of unknown source [75, 76]. The set further contains German strain TS18-98 from minced meat and an historic Mexican strain 6182–50 from a clinical diarrhoea case dating back to 1950 [77]. For the purpose of this study, we used strains EH41 and 4 as reference for ST-820 and ST-223, respectively.

### Genome sequencing, assembly and annotation

Strains were cultured overnight (o/n) at 37 °C with shaking in lysogeny broth (LB) (Fisher Scientific, Thermo Fisher Scientific, Asheville, NC, USA). Total genomic DNA was extracted from o/n cultures using the QIAamp DNA Mini Kit (Qiagen, Inc., Valencia, CA, USA) according to the manufacturer’s instructions, followed by short-read Illumina sequencing on the MiSeq platform. Paired-end libraries were prepared using either the NxSeq AmpFREE Low DNA Library Kit (Lucigen) with 250 bp read length and sequenced with the MiSeq Reagent kit v2 500-cycle (Illumina), or the KAPA HyperPlus DNA kit (Roche) with 300 bp read length and sequenced with the MiSeq Reagent kit v3 600-cycle (Illumina), following the manufacturer’s guidelines. Illumina reads were assembled with SPAdes [77]. The genomes of ST-223 and ST-820 complex strains 4 and EH41 were sequenced to closure by complementing Illumina short-reads with Oxford Nanopore long-reads on the MinION platform. Genomic DNA was diluted to a concentration of 1.5 µg in 46 µl of nuclease-free water. The library was prepared using the Nanopore 1D Ligation sequencing kit SQK-LSK108 (R9) with the Native barcoding kit EXP-NBD103 according to the manufacturer’s instructions and sequenced on a MinION Mk1B with a SpotON flow cell FLO-MIN107 (R9). Nanopore and Illumina reads were used for hybrid assembly using SPAdes in the careful mode, which includes realignment correction [77]. We further assembled a draft genome of strain 6182–50, for which only reads were available in the NCBI Sequence Read Archive (SRA). Resulting assemblies were QCed with QUAST [78, 79]. The chromosomal and plasmid origins of replication, *oriC* (http://tubic.tju.edu.cn/Ori-Finder/) [80] and *repA*, respectively, were designated as the zero point of the closed EH41 and 4 molecules prior to annotation using the NCBI Prokaryotic Genome Annotation Pipeline (PGAP) [81].

### MLST classification

MLST typing was performed using targeted and whole genome schemas developed for *E. coli* [82, 83]. We determined the Sequence Type (ST) by applying three different schemas as follows: The ST was inferred *in silico* using the Achtman (7-gene) [84] and Pasteur schemas [85] by examining assembled genomes (https://cge.cbs.dtu.dk/services/MLST/) [86]. Through *in silico* and PCR-based-interrogation [59, 60], we inferred the ST according to the Whittam 7- and 15-gene MLST scheme (http://shigatox.net/new/tools/ecmlst.html) [87]. Sequences of the target genes were then queried against the EcMLST database [88]. For NCBI-retrieved genomes for which cultures were not available, alleles and ST were determined *in silico* by BLASTn comparison against the EcMLST database [88]. Assembled genomes were further genotyped in MLST 2.0 (https://cge.cbs.dtu.dk/services/MLST/) [86] and Ridom SeqSphere+ (v6.0.2) [89] to establish a whole genome (wg) MLST phylogeny.

### Comparative phylogenomics

#### Whole genome alignment (WGA) phylogeny

The 20 O113:H21 genomes sequenced in this study along with 15 genomes downloaded from NCBI GenBank (Table S1) were used to construct a whole genome-based phylogenetic tree. The phylogeny was inferred from WGAs using Mugsy (v1.2.3) [90] and RAxML (v4.0) [91] with 100 bootstrap replicates. The tree was visualized in Geneious Prime (v2021.1.1) [92] and decorated with strain-associated metadata in EvolView [93–95].

#### Core genome SNP phylogeny

To compute a SNP phylogeny, we used a custom-built core genome (cg) SNP discovery pipeline described in more detail in [96], implemented on the open-source bioinformatics platform Galaxy [97]. The chromosomal core genome was defined as the set of genic and intragenic regions that are not repeated and do not contain phages, IS elements, plasmid regions, genomic islands or other mobile genetic elements, which evolve at different rates and are not indicative of evolutionary relationships. These regions were determined in the designated closed reference O113:H21 strain EH41 as follows: Repeats with NUCmer (v3.22) [98], prophages with PHASTER [99, 100], and IS elements with ISFinder [101], ISEScan (v1.7.1) [102], and ICEberg (v2.0) [103]. The modular pipeline contains the following workflow steps: (***i*) SNP discovery and typing**. When available, Illumina reads were used for read-based SNP discovery. Reads were aligned to the designated reference with BWA-MEM [104]. The resulting alignments were processed with Freebayes (v1.3.1) [105] with the following threshold settings: mapping quality 30, base quality 30, coverage 10, and allelic frequency 0.75. For contig-based discovery, assemblies were aligned to the EH41 reference chromosome using NUCmer followed by SNP prediction with delta-filter and show-snps distributed with the MUMmer package [98, 106]. The resulting SNP panel for each of the query genomes was used for further processing. (***ii)* SNP validation and filtering**. We used several SNP curation strategies detailed in our previous works [96, 107, 108]: catalogued SNPs from each genome were merged into a single SNP panel and SNPs located within identified excluded regions were removed, as well as low quality alignments or misalignments, non-uniformly distributed regions, and InDels, as previously [108–110]. SNPs were further curated by extracting the surrounding 40 nucleotides (nt) for each predicted SNP in the reference genome, followed by BLASTn of these fragments against the query genomes [111]. SNPs with missing information (‘no hits’) or multiple hits were filtered out, as well as ambiguous nucleotides. (***iii*) SNP annotation and chromosomal distribution**. Allelic status and chromosomal position of SNPs were recorded. To account for the biological relevance of these point mutations, polymorphisms were classified into genic or intergenic by mapping the SNPs to the reference genome. SNP-matrix tables were manipulated with Query Tabular Tool [112]. In addition, we developed a genotyper tool to provide SNP statistics reporting on the number of individual genotypes in the phylogeny. ***(iv)* SNP phylogeny**. The curated panel of high quality SNPs served as basis for phylogenetic reconstruction by maximum parsimony with PAUP (v4.0a163) [113] with a 100 bootstrap replicates. The majority rule consensus SNP tree was visualized in Geneious Prime (v2021.1.1) [92] and decorated in EvolView (v3) [93–95]. Calculation of the consistency index (CI) in Mesquite (v3.6) [114] for each SNP allowed us to identify parsimony informative SNPs and flag homoplastic SNPs, as previously described [96, 107, 108, 115–117]. For genomes retrieved from NCBI GenBank, where reads were not available, we interrogated the allelic status of the catalogued SNPs in the assembled genomes.

#### Whole genome MLST derived phylogeny of STEC O113:H21

Classification results from the wgMLST analysis in Ridom SeqSphere+ [89] were used to construct a minimum spanning tree (MST) for alleles present in all isolates with default settings. The resulting tree was decorated with strain-specific metadata, including the respective allele status of the 7-gene and 15-gene Whittam MLST classification [87] inferred from EcMLST [88].

#### Pathogenome make-up of STEC O113:H21

The virulence complement was identified using VirulenceFinder (https://cge.cbs.dtu.dk/services/VirulenceFinder/) [118, 119] and VDFDB [120]. The resistome was analysed with the Comprehensive Antibiotic Resistance Database (CARD) (https://card.mcmaster.ca/home) [121], ARG-ANNOT [122] and ResFinder (https://cge.cbs.dtu.dk/services/ResFinder/) [123, 124]. Prophages were distinguished from the core genomes using PHASTER [99, 100]. Plasmid replicon and relaxase types and conjugation potential was determined with PlasmidFinder (https://cge.cbs.dtu.dk/services/PlasmidFinder/) [125] and MOB-suite [126].

#### Stx-bacteriophage profiling and visualization

Boundaries and locations of intact, partial, or remnant prophages were identified using PHASTER [100]. For Stx-bacteriophages the stx-subtypes and insertion sites were recorded as described in (Scheutz *et al.*, 2012; Ashton *et al.*, 2015). The *in silico* delineated *stx* subtypes for the O113:H21 strains sequenced in this study, were confirmed by PCR according to (Scheutz *et al.*, 2012) using GoTaq Green Master Mix (Promega) in a 25 µl reaction volume. The Stx-bacteriophages of closed genomes were compared and visualized using Geneious Prime (v2021.1.1) [92] and Easyfig (v2.2.2) [127].

#### Shiga toxin subtyping

To confirm the *stx_2d_* allele subtype *in silico*, complete *stx_2_* genes were translated and aligned using Clustal Omega (https://www.ebi.ac.uk/Tools/msa/clustalo/) [128, 129]. We confirmed the presence of the activatable property of Stx_2d_ located at the C-terminal end of the A_2_ subunit (KSQSLYTTGE). Amino acid sequences of the B-subunit of Stx_2d_ are distinguished from Stx_2a_ by serine (S) and glutamic acid (E) along with the N-terminal END-motif motif [130, 131]. Virulence genes were identified with VirulenceFinder [118, 124, 132] and VFDB [120].

#### Mobile genome element (MGEs) and InDels

Insertion sequence elements (IS) were identified and classified with Iceberg [103], and ISEScan [102] in Galaxy [133]. ICE/IME regions were determined in ICEfinder (https://bioinfo-mml.sjtu.edu.cn/ICEfinder/index.php) [103]. Genomic islands (GI) were detected with IslandViewer4 [134–136]. InDels between the closed chromosomes of strains EH41 and 4 were identified in Geneious Prime (v2021.1.1) [92].

#### Comparison of genome architectures and distribution of virulence genes

Architectures and gene inventories of closed chromosomes and virulence plasmids were comprehensively analysed and visualized with Blast Ring Image Generator (BRIG) [137]. Chromosomal, phage-and plasmid-borne pathogenicity genes were recorded with Virulence Finder [118, 124, 132], PHASTER [100] and by tBLASTn [111] query of a list of established O113:H21 virulence determinants [56, 60]. To study the prevalence of identified virulence gene complement of the core and carried plasmids in all sampled closed and draft genomes, we used Large-Scale blast Score Ratio (LS-BSR) [96, 138, 139] with tBLASTn [111]. We compared both the overall proteome inventory and the prevalence of the combined virulence factor complement identified in this strain set. For each of the proteins, a BLASTp raw score was obtained for the alignment against itself (reference bit score) and the most similar protein (query bit score) in each of the genomes. The BSR value was calculated by dividing the query bit score by the reference bit score, resulting in a BSR value between 0.0 and 1.0. Proteins with a normalized BSR of <0.4 were not considered homologous. The resulting BSR matrix or alternatively the percent identities were visualized as heatmaps with Multiple Experiment Viewer (MeV) (v4.8) [140] and Morpheus (https://software.broadinstitute.org/morpheus).

#### Stx-production pathotypes

The Stx_2_-production phenotypes of the cultures were determined by recording the Stx litres through Enzyme-Linked ImmunoSorbent Assay (ELISA) under both spontaneous and Mitomycin C (MMC)-induced conditions. Overnight (o/n) cultures were diluted to an OD_600_ of 0.05 and grown to an OD_600_ of 0.3–0.5 in fresh LB media at 37 °C. At this stage, cultures were split and incubated at 37 °C for 6 h under non-induced and induced (MMC: 0.5 µg ml^−1^) conditions. Toxin production was measured after harvesting 5 ml of each culture for parallel processing. To lyse bacterial cells and release produced Stx, induced cultures were treated with polymyxin B (6 mg ml^−1^ 37 °C, 10 min). Supernatants were collected after centrifugation (3500 r.p.m., 10 min), filtered through 0.22 µm low protein-binding membrane filters (Millex-GP; Millipore) and diluted to measurable concentrations. Stx_2_-production was measured using the Premier EHEC kit (Meridian Bioscience, Cincinnati, OH) following the manufacturer’s instructions. Titres were calculated using a standard curve generated from serial dilutions of purified Stx_2a_ (BEI resources, NR-4478). Statistical significance was determined using Prism v9.0.1 (GraphPad Software, San Diego, CA). A two-way ANOVA with Sidak’s multiple comparisons test was used to compare non-induced vs. MMC-induced conditions for each strain. A one-way ANOVA with Tukey’s multiple comparisons test was used to compare Stx-production by ST-223 and ST-820 groups under (both non-induced and MMC-induced conditions.

## Results

### Whole genome sequencing of a global collection of 20 O113:H21 strains

For this study, we sequenced and analysed the pathogenomes and Stx-production traits of 20 O113:H21 isolates sourced from the bovine reservoir, produce, or clinical cases (Table S1); along with 15 O113:H21 genomes retrieved from NCBI GenBank, which added four closed and 11 draft genomes to our dataset [64, 75, 76]. The strains in our collection represent the two major phylogeographical complexes: Australian ST-820 and ST-223 strains from the US and elsewhere including subvariants (Table S2) (Reid *et al.*, 2000). To establish a refined high-resolution phylogenomic framework for this lineage, we sequenced the genomes of the designated reference strains EH41 and 4 to closure using the Nanopore LRT platform. These strains served as high quality representative genomes for the ST-820 and ST-223 complex respectively, while the other strains were sequenced using Illumina technology to draft stage, yielding between 41 and 131 contigs (Table S1). The predicted genome sizes in this set range from 4.9 to 5.1 MB with an average GC-content of 50.8 %. The chromosomes of strains EH41 (ST-820) and 4 (ST-223) are: 5 040 503 and 4 907 913 bp in size with 4978/5054 coding sequences (CDS), 89/94 tRNAs and 22/22 rRNAs, respectively. The application of LRT allowed us to also recover and accurately assemble the plasmids of strains 4 and EH41 (Table S1). MLST types, as inferred from the 7- and 15-gene Whittam schemes, grouped the isolates into two major phylogeographical complexes (Table S2) [59, 60]: ST-820 strains, a distinct clonal group found in Australia, and ST-223 strains found in the US [55, 64] and elsewhere in the world [65–67]. However, two US strains from ground beef, 16 and 53, were classified as ST-820, characteristic for Australian strains. A likely explanation is that the US imports lean boneless beef trimmings from different countries as ingredients for ground beef manufacturing. Strain 53, was isolated in the US from such trim that had been imported from Australia [73]. Although direct production records are lacking, strain 16 was isolated from ground beef later produced by the same supplier of the imported beef trim samples [68], thus strain 16 too, likely originated in Australia. All but two non-Australian strains are of ST-223, while clinical isolates 2013 C-3181 and 10 are classified as ST-846 and ST-234, respectively.

### Comprehensive analyses of pathochromosome architectures, phages and plasmids

**Fig. 1.**
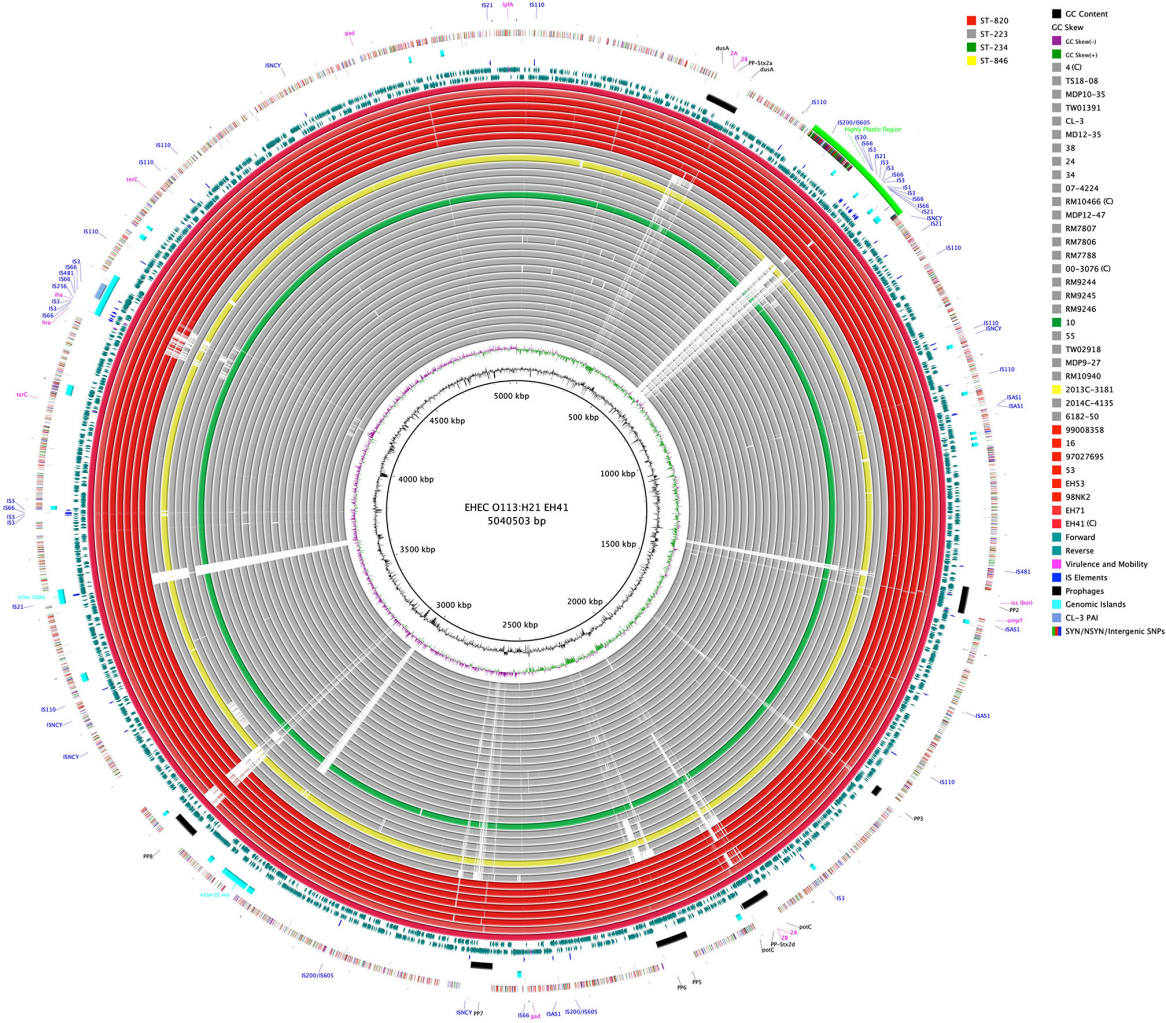
Chromosome architecture of STEC O113:H21 BRIG comparison of O113:H21 genomes referenced to the 5 040 503 bp chromosome of ST-820 strain EH41. CDS are presented as arrows on the +/-strands, and functional annotations for virulence genes and other loci of importance are highlighted as shown in the figure legend. Query genomes are color-coded according to ST and the order plotted in the circle reflects their respective phylogenetic positions. Chromosomal synteny is disrupted by multiple prophages and other MGEs. GC-content and GC-skew of the EH41 chromosome are depicted in the two innermost circles. ^(C)^ denotes closed chromosomes.

**Fig. 2.**
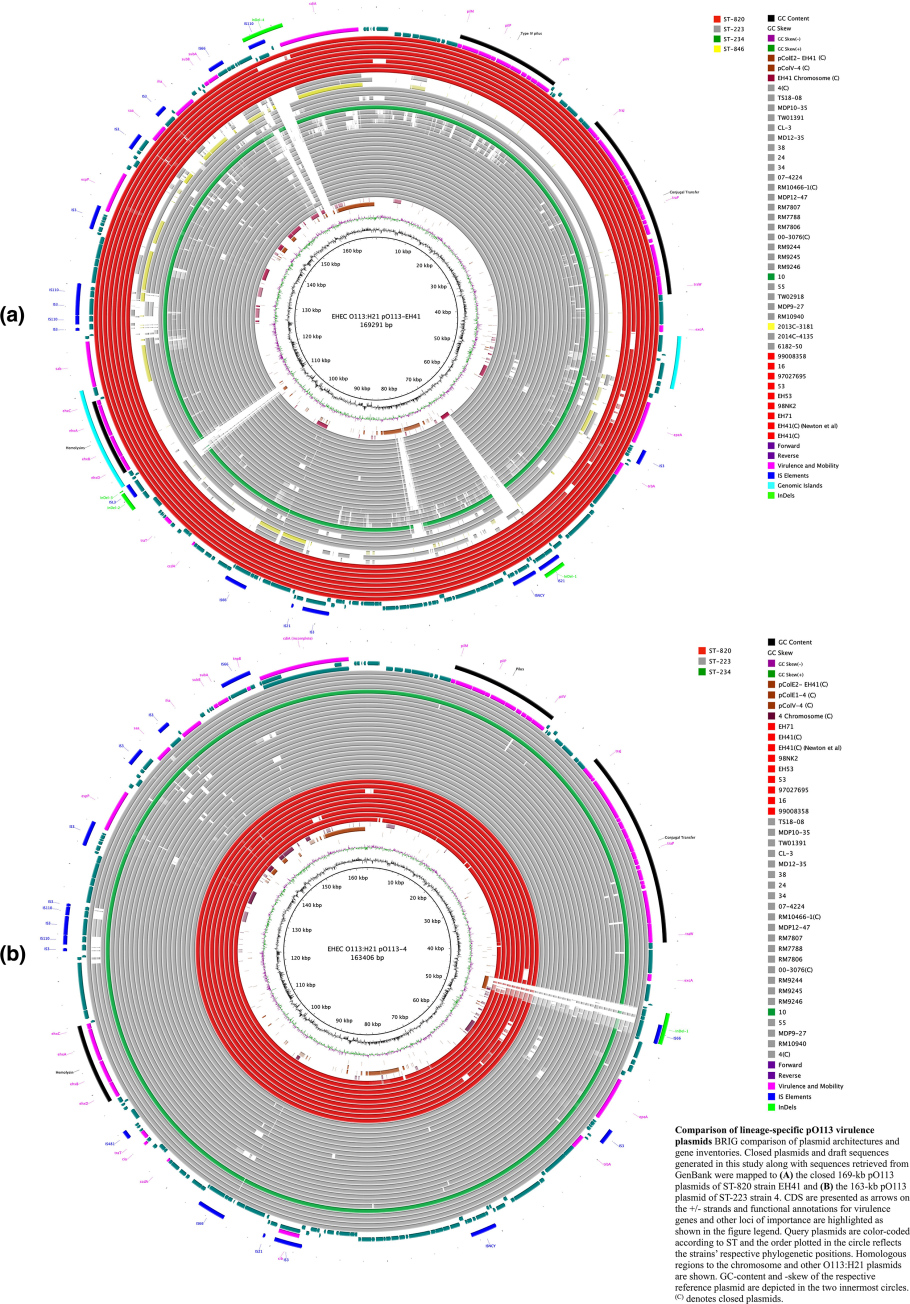
Comparison of lineage-specific pO113 virulence plasmids BRIG comparison of plasmid architectures and gene inventories. Closed plasmids and draft sequences generated in this study along with sequences retrieved from GenBank were mapped to (**a**) the closed 169 kb pO113 plasmids of ST-820 strain EH41 and (**b**) the 163 kb pO113 plasmid of ST-223 strain 4. CDS are presented as arrows on the +/-strands and functional annotations for virulence genes and other loci of importance are highlighted as shown in the figure legend. Query plasmids are color-coded according to ST and the order plotted in the circle reflects the strains’ respective phylogenetic positions. Homologous regions to the chromosome and other O113:H21 plasmids are shown. GC-content and -skew of the respective reference plasmid are depicted in the two innermost circles. ^(C)^ denotes closed plasmids.

**Fig. 3.**
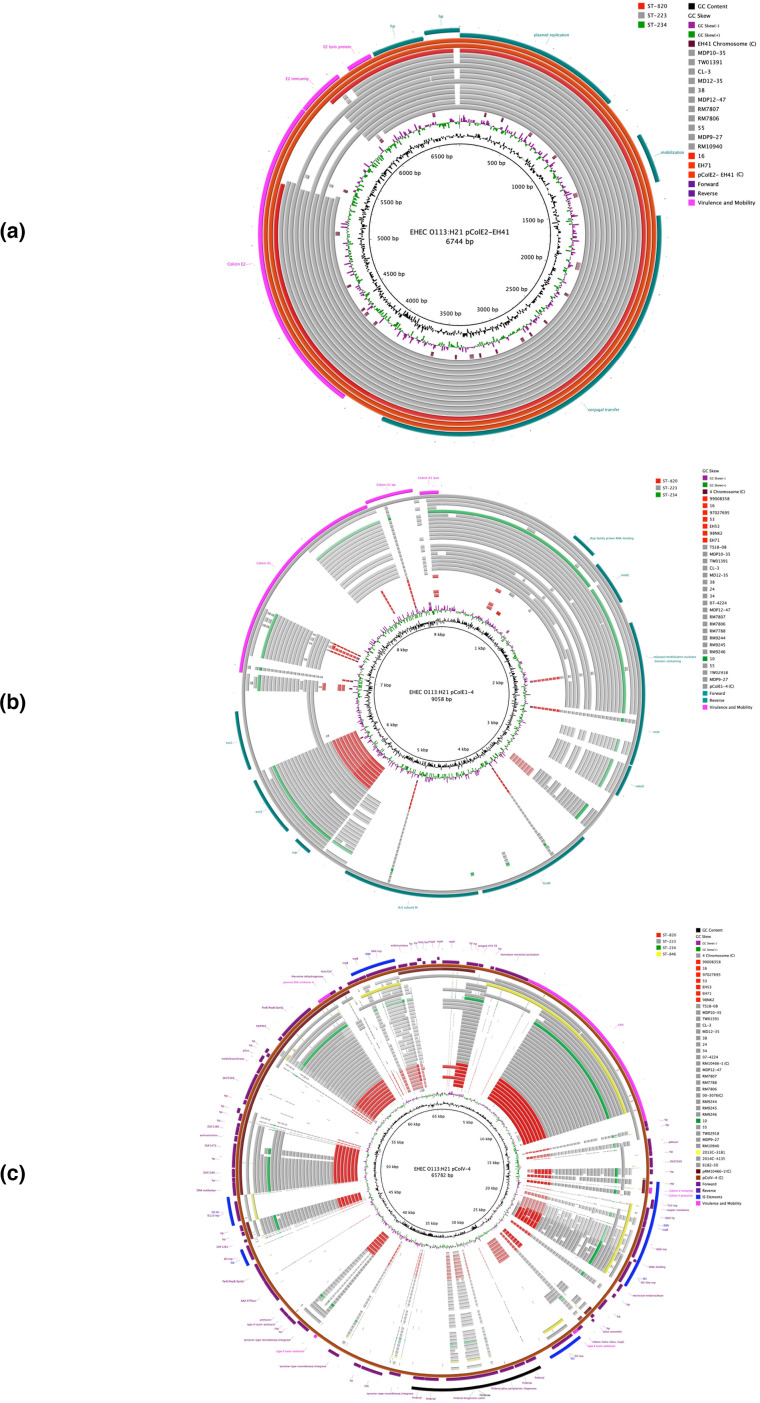
Colicin plasmids in closed reference strains EH41 (ST-820) and 4 (ST-223) BRIG comparison of colicin plasmid architectures and gene inventories of (**a**) pColE2-EH41, (**b**) pColE1-4 and (**c**) pColV-4. CDS are presented as arrows on the +/-strands and functional annotations for virulence genes and other loci of importance are highlighted. Query plasmids are color-coded according to ST and the order plotted in the circle reflects the strains’ respective phylogenetic positions. Homologous regions to the chromosome and other O113:H21 plasmids are shown. GC-content and -skew of the reference plasmid are depicted in the two innermost circles. ^(C)^ denotes closed plasmids.

To initially assess the degree of chromosomal plasticity within the O113:H21 serotype we compared the closed chromosomes of ST-820 and ST-223 reference strains EH41 and 4 along with four closed genomes retrieved from GenBank (2014C-4135 (ST-223), 00–3076 (ST-223), 2013 C-8131 (ST-846) [76] and RM10466 (ST-223) [74]. The pairwise identity of ST-820 strain EH41 to strains 4 (ST-223) and 2013 C-8131 (ST-846) is 79.6 and 66.6 %, respectively. The chromosomal architectures of closed and draft genomes were compared using strain EH41 (ST-820) as the designated reference. As evident in Fig. 1, we observed a largely genome-wide synteny of the chromosomal backbone disrupted by multiple mobile genome elements (MGE), which are major contributors of O113:H21 genome diversification [141]. Catalogued prophages and their respective length, chromosomal insertion sites, GC-content, and number of predicted coding sequences (CDS) are listed in Table S3. In the six closed genomes that were available for this study, prophages account for 5.5–6.1 % of the total chromosome and their dynamic acquisition and secondary loss contributes to the genome size variation observed in the analysed strains (Table S1). We further identified a highly plastic region (HPR) spanning about 200 kb that features multiple InDels associated with MGEs. A comparison referenced to strain 4 (ST-223) can be found in Fig. S1. We note that the phylogeographical separation of ST-223 and ST-820 complex isolates is mirrored in the HPR genomic organization and composition. The genomes of the closed ST-223 strains 4, 00–3076, and RM10466 are distinguished by deletions not present in EH41 (ST-820). This particular pattern was also seen in the remainder of analysed ST-223 and ST-820 draft genomes with the notable exception of ST-223 strains 6182–50 and 2014 C-4135. The lineage-specific virulence plasmid, pO113, was detected in the assemblies of all but four strains (ST-223 strains 6182–50, 2014 C-4135, TW02918 and ST-846 strain 2013 C-3181) [141] (Fig. 2). The four closed plasmids shows a highly conserved and syntenic plasmid backbone without any major structural differences featuring a 99 % nucleotide identity over the entire plasmid length [111, 142]. Our analysis identified several InDels when compared to the closed ST-reference pO113 plasmids of strains EH41 and 4 (Fig. 2), which are associated with mobile genetic elements (Fig. S2). The genetic relatedness of strains is also reflected in the plasticity found within the lineage-specific virulence plasmid (Fig. 2). We note here that we identified several plasmid-borne signatures unique to ST-820/ST-223 that can be utilized for ST genotyping in analogy to chromosomal MLST markers defining the ST. Colicins are produced by and are toxic to some *E. coli* strains to reduce competition from other phylogenetically related microbial strains [143], including certain O113:H21 strains [143, 144]. Both closed ST-reference EH41 and 4 strains are colicinogenic and code for colicins E2 and E1, respectively encoded on relative small plasmids [143] (Fig. 3). Strain 4 further carries a 65 782 bp colicin V plasmid pColV-4 [145]. BLASTn of its sequence [111, 142] against the non-redundant (nr) NCBI database revealed local similarity to O113:H21 plasmid pRM10466-2 (Fig. 3c) and other phylogenetically diverse *E. coli* plasmids. Plasmid profiling of the other genomes identified a number of plasmid replicons, which provides a testament to the considerable plasticity of the plasmid types carried in this lineage. Further details on replicon subtypes and plasmid content can be found in Table S4, although the draft status of these genomes did not allow us to fully reconstruct their plasmid inventories.

### High-resolution phylogenomic framework for STEC O113:H21

**Fig. 4.**
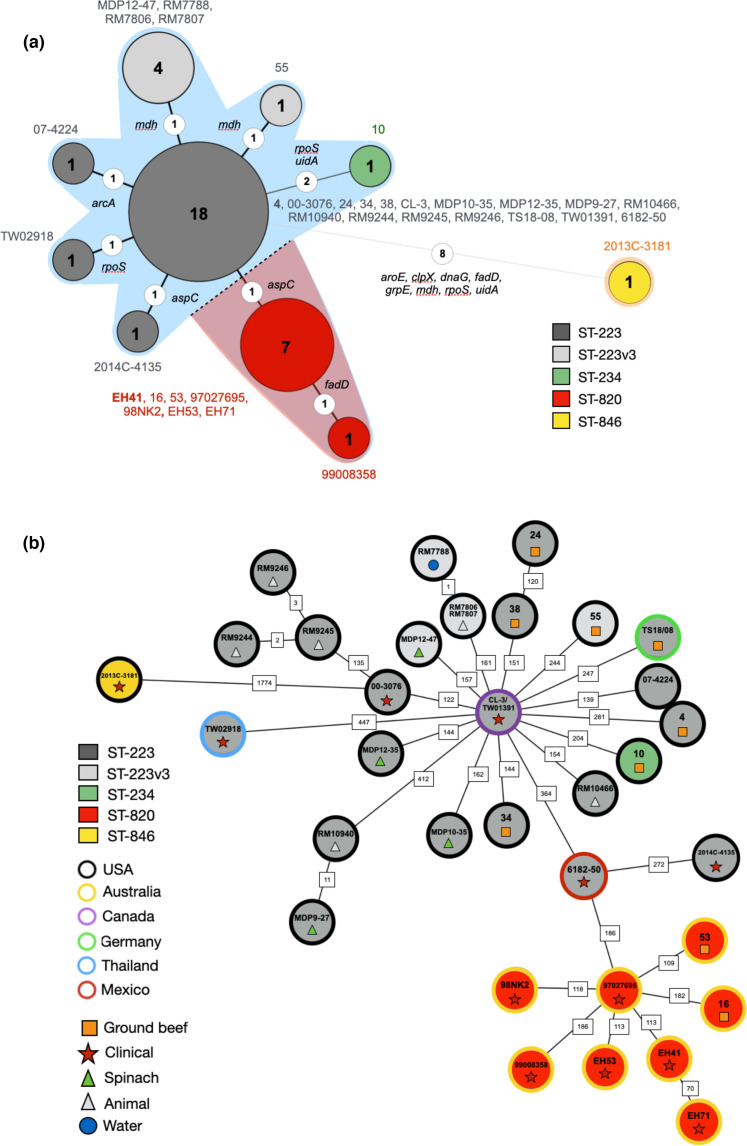
Minimum Spanning Tree based on MLST analyses. (**a**) The MLST phylogeny for 35 O113:H21 strains based on the 15-gene Whittam schema was constructed in Ridom SeqSphere+ [89]. Circle size corresponds to the number of isolates with the same allele status (Table S2). Numbers on connecting branches show allelic differences between strains. (**b**) wgMLST-based phylogeny. The closed chromosome of isolate EH41 (ST-820) was provided as a seed for wgMLST analysis in Ridom SeqSphere+ [89]. The shared gene inventory of the 35 sampled O113:H21 genomes was determined at 3559 genes. The established wgMLST phylogeny clearly reflects the phylogenomic, as well as geographical separation of Australian ST-820 from ST-223 isolates found elsewhere and provides further evidence for a quasi-intermediate state of the Mexican strain 6182–50, positioned between the two major complexes. Distance values represent the number of genes with differing allele status in the network. Circle colours denote ST-classification according to the 7-gene Whittam MLST scheme [87]. Stars indicate the clonal status of strains TW01391 and CL-3, which were derived from the same repository and, as expected, are found clustered [59].

**Fig. 5.**
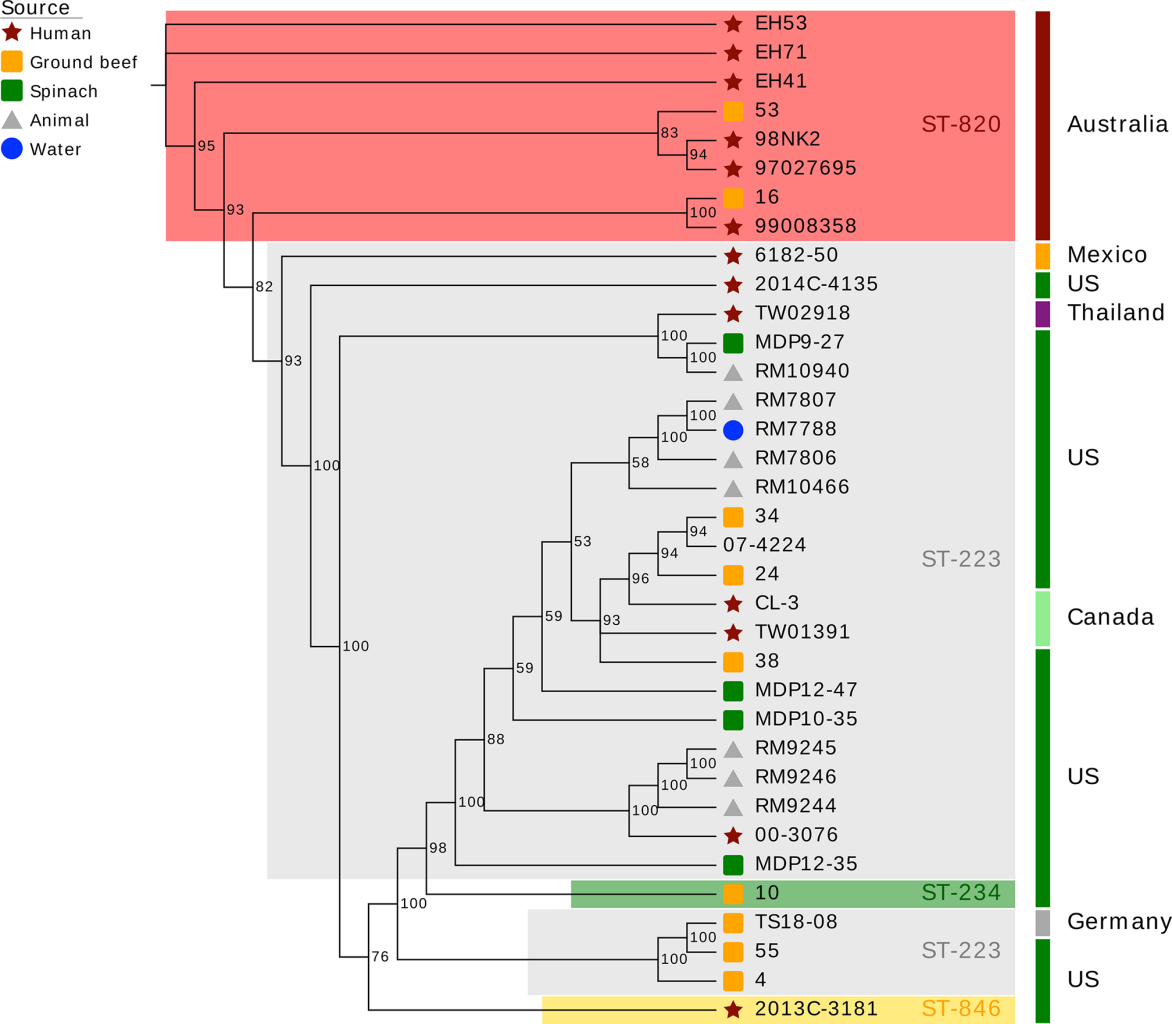
Whole genome alignment phylogeny. Genomes of a total of 35 isolates, comprised of 20 strains sequenced in this study along with 15 strains obtained from NCBI GenBank, were aligned with Mugsy [90]. The phylogenetic tree was inferred using RAxML [91] with 100 bootstrap replicates and decorated with bootstrap support and other strain-associated metadata in EvolView [93, 95]. The tree topology partitions the isolates into two major phylogeographical complexes, separating Australian ST-820 from ST-223 strains found in the US and elsewhere, and further, suggests a quasi-intermediate position of the Mexican ST-223 strain 6182–50.

**Fig. 6.**
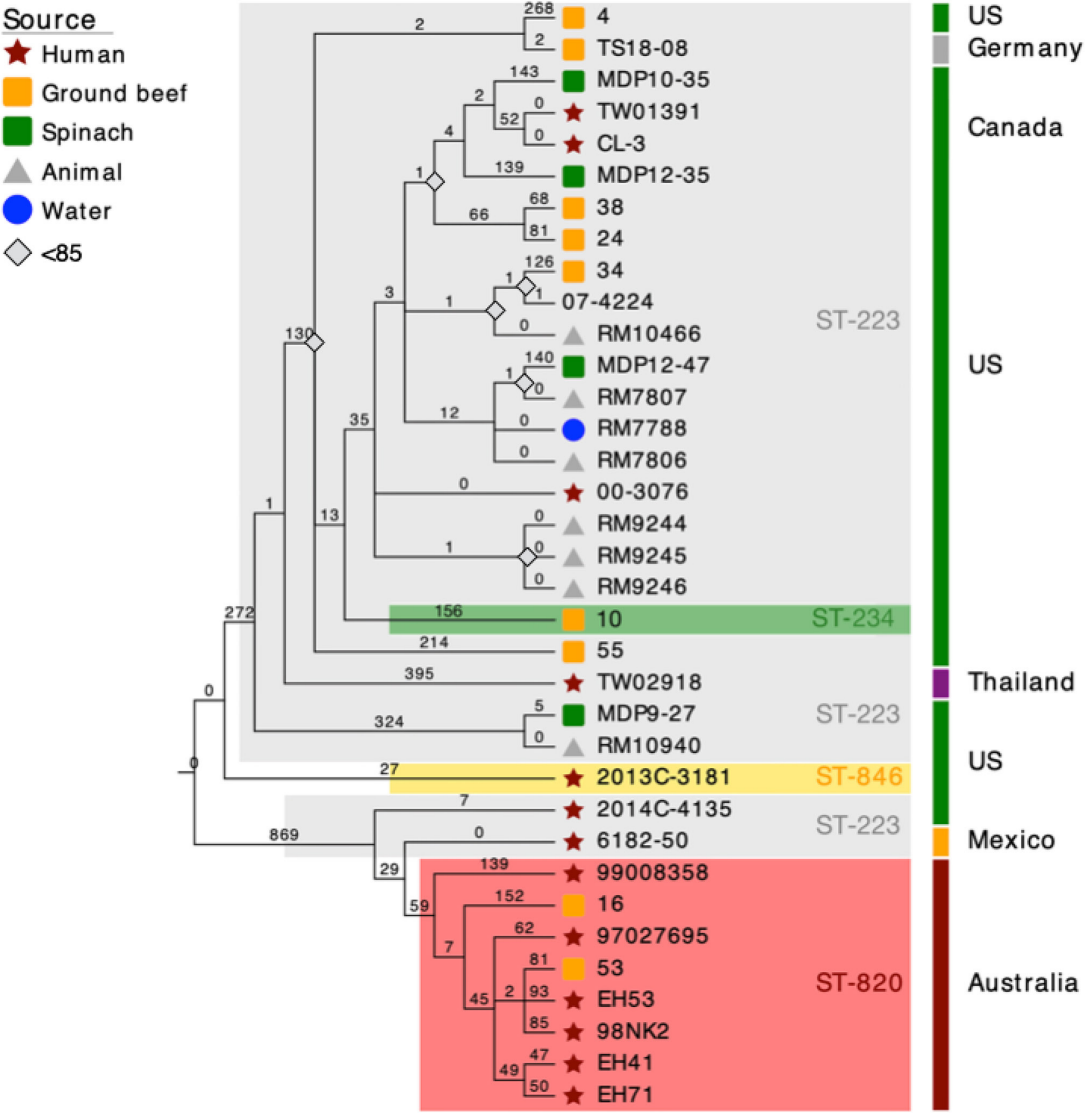
Maximum parsimony cgSNP-phylogeny. Comparison of 35 genomes yielded a total of 4398 SNPs of which 1979 were parsimony informative. The tree shown is a majority-consensus tree of 3010 equally parsimonious trees with a CI of 0.99, decorated with strain-associated metadata in EvolView [93, 95]. Trees were recovered using a heuristic search in PAUP (v4.0a163) [113] with 1000 bootstrap replicates. Bootstrap supports below 85 as well as numbers of separating SNPs are shown. The tree topology mirrors the established MLST- and WGA-phylogenies, separating ST-820 from ST-223 complex isolates.

To investigate the phylogenomic boundaries between ST-820 and ST-223 and to determine the relationships of individual strains, we established a refined high-resolution phylogenomic framework for the O113:H21 lineage [60, 67] that was inferred from expanded wgMLST, WGA, and SNPs. We computed an MLST phylogeny based on the Whittam schema (15-gene) by inferring the allele status in Ridom SeqSphere+ and assigning allele profiles in the EcMLST database. (Fig. 4a) [88, 89]. Within ST-223 and ST-820 complex isolates we detected further allelic variations when applying both the Whittam and 7/15-gene MLST schemas, which is detailed in Table S2. Five strains (RM7788, RM7806, RM7807, MDP12-47 and 55) are classified as ST-223v3, distinguished by alleles *mdh8* ^C124T^ or *mdh318* ^G526T^ [59]. The wgMLST phylogeny, decorated with the delineated ST-types, mirrors the phylogeographical separation observed in the MST tree, but provides higher resolution (Fig. 4b). Two ST-223 strains, 6182–50 from Mexico [77] and 2014 C-4135 from the US, are positioned in the phylogenetic boundary between the major ST-223 and ST-820 complexes, suggesting that these isolates might occupy a quasi-intermediate position. To further investigate this question, we constructed phylogenetic hypotheses that are based on whole genome alignments (WGA) and *de novo* SNP discovery [96]. The resulting WGA tree topology (Fig. 5) corroborates with phylogenetic placement of strains by wgMLST (Fig. 4). Core genome (cg) SNPs in the 35 O113:H21 genomes yielded a total of 4398 high-quality SNPs (Table S5). The constructed maximum parsimony (MP) tree shows bootstrap supports greater than 80 % for the majority of nodes (Fig. 6), and its topology mirrors the generated wgMLST- and WGA-phylogenies (Figs 4 and 5). In the catalogued SNP panel, we identified 1979 parsimony informative SNPs and delineated a total of 77 clades and clusters differentiating individual SNP genotypes (Table S6). These genotypes represent about three times the number of tree nodes (#25) in the tree, which is attributed to the high number of terminal strain-specific SNPs. Overall SNPs were found dispersed throughout the EH41 reference chromosome; however, we noted an elevated SNP density (Fig. S3) in the previously identified region of high plasticity (Fig. 1). The ST-223 and ST-820 complex isolates are distinguished by their particular SNP pattern within this highly plastic region. The majority of genes in this area feature relatively high SNP numbers considering gene length and fulfil diverse functions, such as in metabolic pathways, substrate utilization, and stress response (Table S5), which may imply a potential role in the phylogeographical diversification of O113:H21. The majority of genic SNPs of this locus are synonymous (88.4 %) that may suggest evolutionary pressure. However, a total of 52 genes in this region feature non-synonymous SNPs (Table S8). Among these are the translocation and assembly module (*tam*) for secretion of adhesins [146] and metabolic operons, such as LaAscorbate dissimilation (*ula*) [147], *ytf* and trehalose (*tre),* along with the *fim* operon. The latter four loci were previously identified as SNP hotspots in extraintestinal pathogenic *E. coli* of ST-131 [148]. Such accumulation of regionally localized SNPs may indicate mutational hotspots [109] or, alternatively, suggests a potential site of recombination. As a result of these scenarios, the phylogenetic signal can be in conflict with the signal from clonally inherited regions. Maximum parsimony (MP) provides a homoplasy metrics as indicator of accuracy and also a basis to identify potential recombination events [149, 150]. The calculated overall consistency index (CI) [114] of the tree is 0.99, and we found no evidence that SNPs in this HPR region are biassed towards homoplastic (CI=<1) or multiallelic SNPs (Table S7), which are not confined to a particular region and found scattered throughout the chromosome. Homoplasy may simply be the result of random nucleotide substitutions over time and thus may not require an evolutionary explanation. The SNP signature within the HPR allows distinguishing ST-223 from ST-820 complex isolates (Fig. 1) with the notable exception of ST-223 strains 6182–50 and 2014 C-4135. Their architecture and SNP profile in this particular region rather resembles the ST-820 complex (Table S5). In fact, when using the Achtman MLST scheme (Table S2), both strains grouped with the Australian clonal complex, a finding that is in agreement with the strain’s supposed quasi-intermediate phylogenetic position (Figs 4–6). The phylogeographical separation into the two major ST complexes is also evident in the comparison of the proteome inventories by hierarchical average linkage clustering (Fig. S4).

### Comprehensive analyses of Stx-status and virulence genes

**Fig. 7.**
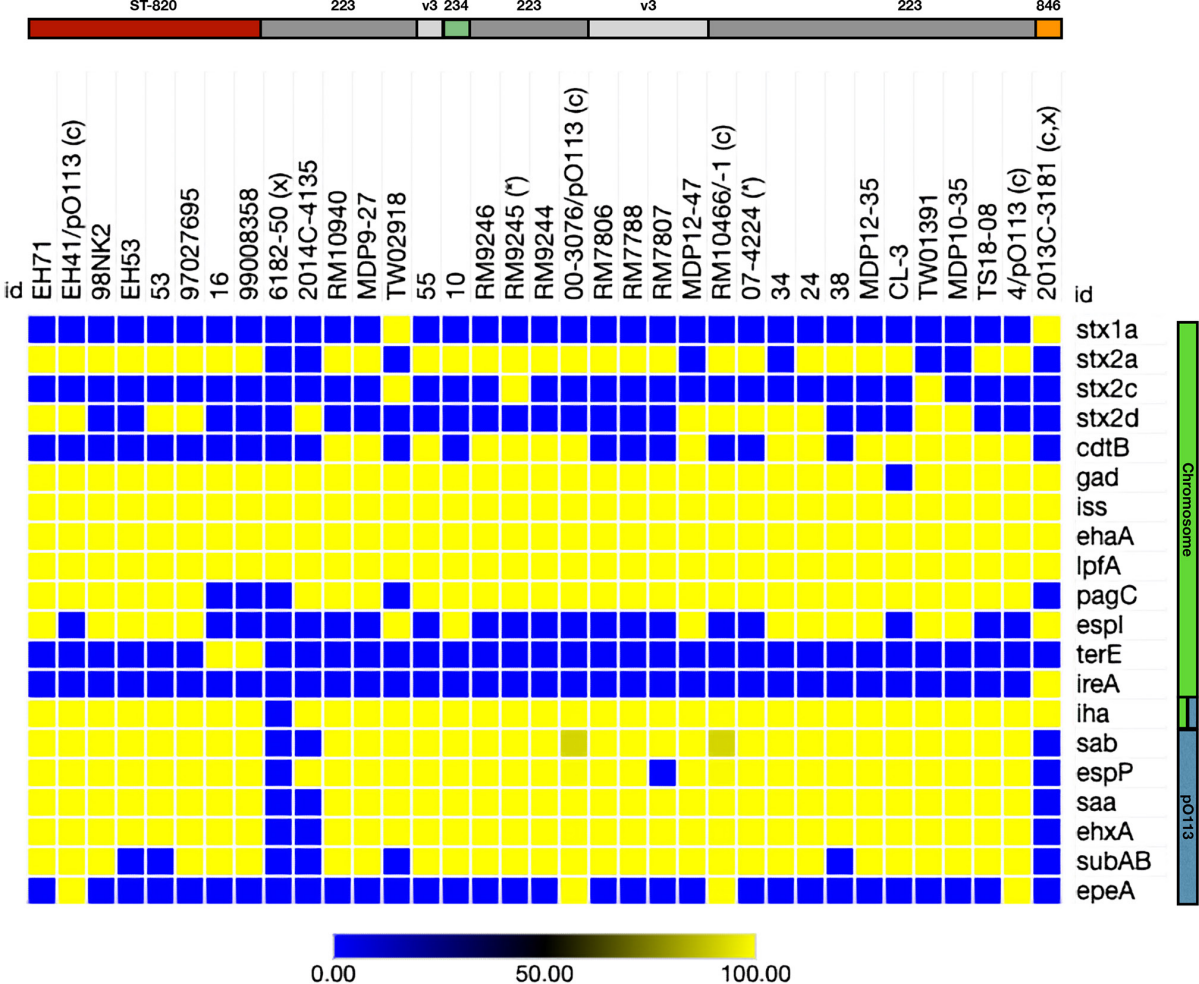
Virulence gene inventory. The heatmap is based on the nucleotide percentage identity of virulence genes. Prevalence was determined by BLASTn query of these loci with a minimum 60 % length requirement. Percentage identities for each gene are visualized in a heatmap showing their prevalence and distribution using Morpheus (https://software.broadinstitute.org/morpheus). The order of strains on top reflects their inferred phylogenomic position. The coloured bar on the left indicates the coding molecule. ^(C)^ denotes a closed genome; ^(x)^ pO113 not detected or sequence not available; and ^(*)^ fragmented *stx*-loci in the draft genomes of strains 07–4224 and RM9245 prevented determination of the suballele *in silico*. None of the 35 analysed strains carry antibiotic resistance genes [123], an observation in agreement with previous studies [209, 210]. Details on prevalence can be found in Table S8.

**Fig. 8.**
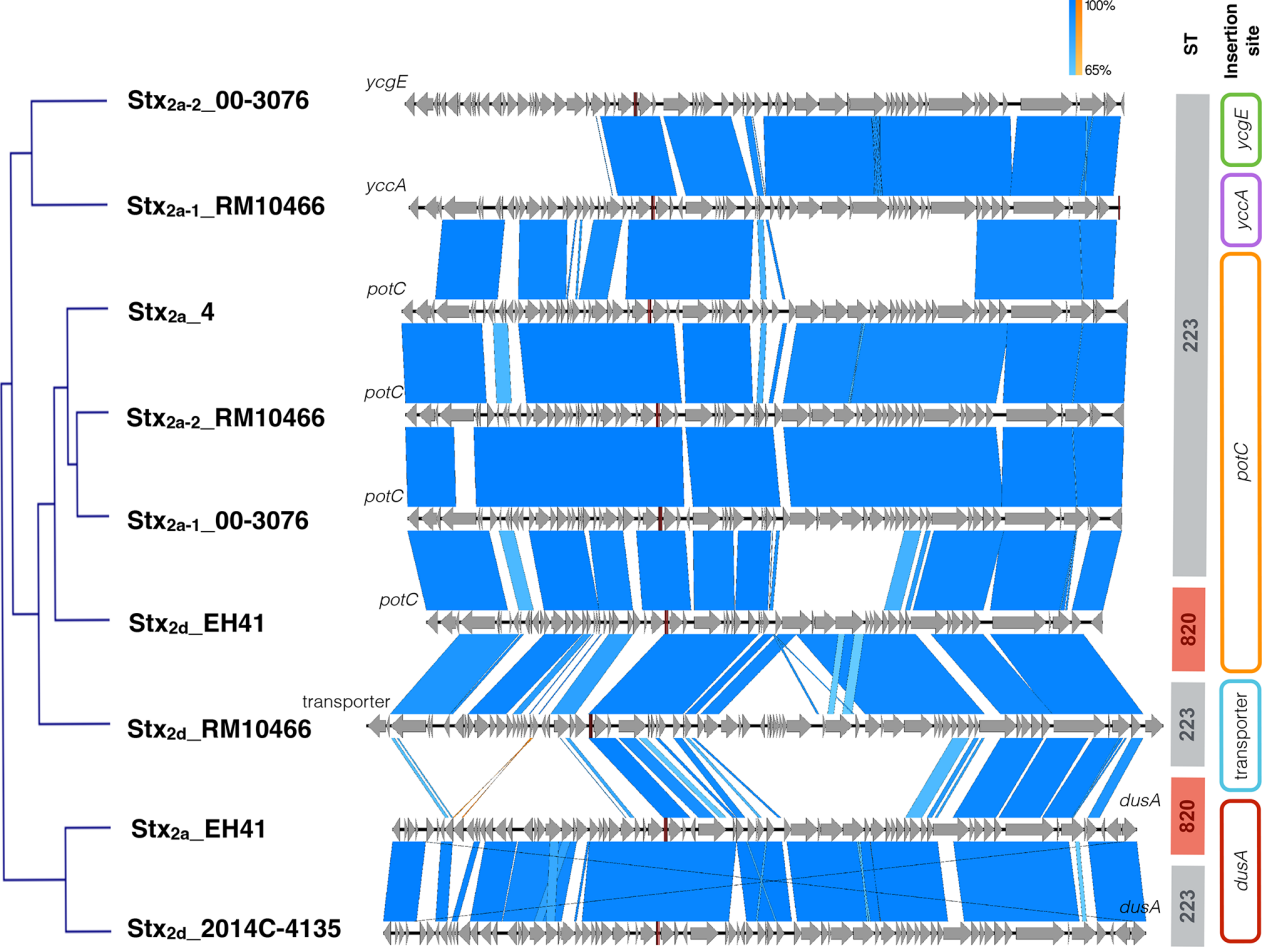
Stx_2_-prophage inventory. Stx_2_-phage architectures were compared by BLASTn and visualized in Easyfig [127]. Phage CDS are depicted as grey arrows. Blue homologous blocks indicate unidirectional sequence similarity. The *stx*-suballele along with the respective chromosomal insertion and MLST of the bacterial host strain is shown.

The prevalence of the identified chromosomal, phage-and plasmid-borne virulence genes revealed a considerable plasticity in the strains’ individual virulence complement, though we did not detect a clear-cut virulence profile boundary that would allow to distinguish ST-223 from ST-820 complex isolates (Table S8). Carriage of Stx-prophages is a virulence hallmark of STEC; the Mexican strain 6182–50 however is distinguished from all the other analysed strains by its *stx*-negative status featuring intact Stx-phage insertion sites in its draft genome. Such atypical STEC-like isolates have been described in a number of lineages. Such strains either never acquired Stx-phages or may have secondarily lost *stx* during the course of infection, isolation or routine subculture [117]. The remainder of strains carry up to three Stx-phages of different or the same *stx*-subtypes (Fig. 7) featuring suballeles *stx_1a_*, *stx_2a_*, *stx_2c_* and/or *stx_2d_* (Table S8) [130]. The major *stx_2_* allele was found in 33 strains, and two strains carry *stx_1_* alone (2013 C-3181) or in combination with *stx_2c_* (TW02918). Except for strain 07–4224, for which only a draft sequence was available [75], the *stx_2_* suballele could be unambiguously identified either by *in silico* analysis or PCR interrogation. In 74 % of the 35 strains analysed we detected the most cytotoxic subtype *stx_2a_* [151]; a comparison of Stx_2a_-phages is shown in Fig. 8. This suballele is present in all eight sampled Australian ST-820 strains but absent in seven of the 35 ST-223/234/846 strains we examined. Stx_2a_ is carried either alone (13/35), or in combination with another copy of Stx_2a_ (7/35), Stx_2c_ (1/35) or Stx_2d_ (5/35). Strain RM10466 is the only isolate that carries three Stx-prophages (Stx_2a_ x2 and Stx_2d_). We further noticed that all but one of the clinical HUS isolates are *stx_2a_*-positive, with the exception of ST-223 strain TW01391 (*stx_2c_*, *stx_2d_*). In the analysed strain set, Stx-phages are inserted at six different sites, some of which are established phage target sites in STEC (Table S8): Phage Stx_1a__2013 C-3181 is inserted into *wrbA*, as described before [24, 40, 152, 153]. Four Stx_2_-phages (Stx_2a__4, Stx_2a-1__00–3076, Stx_2a-2__RM10466, and Stx_2d__EH41) disrupt the spermidine uptake operon *potAB-CD* [154, 155]. In analogy, *potC* was previously found to be occupied by O113:H21 Stx_2a_-phages [64], an O2:H25 Stx_2g_-phage [3, 153], as well as other O157:H7 Stx-phages [4, 107]. Stx_2_-phages in EH41 (Stx_2a_) and 2014 C-4135 (Stx_2d_) are both inserted at *dusA*. This tRNA-dihydrouridine synthase also carries a phage scar in O157:H7 strain EC4115 [107]. Other Stx_2_-phages are located between two transporters (Stx_2d__RM10466), downstream of the HTH-type transcriptional repressor *ycgE* (Stx_2a-2__00–3076), or the BAX-inhibitor *yccA* gene (Stx_2a__RM10466), the latter associated with the Sp4-prophage in O157:H7 [4, 107, 156]. Phages Stx_2a__EH41 and Stx_2d__2014 C-4135 feature different *stx*-alleles though show overall structural similarities and are both inserted into the tRNA-dihydrouridine synthase (*dusA*). We noted that the four phages inserted at *potC* (Stx_2a__4, Stx_2a-2__RM10466, and Stx_2a-1__00–3076 and Stx_2d__EH41) all feature the same highly homologous integrase type. Taken together, our data did not show an association between Stx-phage subtypes and insertion sites [153], but may suggest that the sequence specificity of the respective integrase type determines chromosomal phage location, independent of its featured *stx_2_*-suballele [157, 158]. Stx_2_-phages were likely subjected to recombination events that may have led to an exchange of the *stx*-allele, in analogy to a potential antiterminator shuffling [107], while other phage characteristics were maintained, and resulted in an overall mosaic-like phage composition, as observed in other LEE-negative STEC [153]. The prevalence of *stx* subtypes (and associated phages), does not necessarily reflect the core genome relationships (Figs 4–6), as a strain’s MGEs inventory is also shaped by environmental niche, independent of Stx-phage acquisition, rather than by a common evolutionary history [159, 160].

### Stx_2a_-production by ST-820 and ST-223 complex isolates

**Fig. 9.**
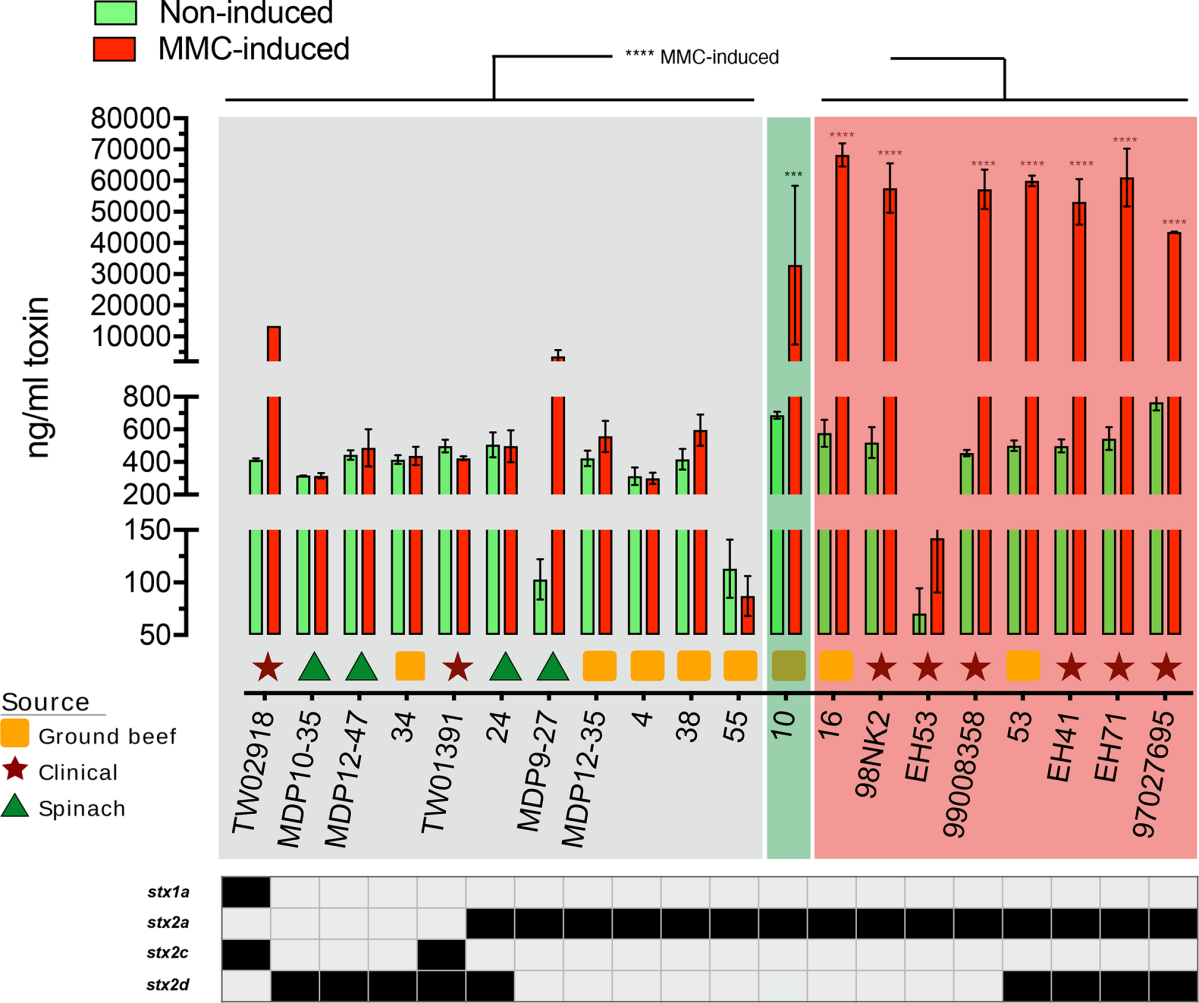
Variability in Stx-production. Stx_2_-litres of ST-820 and ST-223 cultures were determined by ELISA under non-induced and MMC-induced conditions. A two-way ANOVA with Sidak’s multiple comparisons test was used to compare non-induced vs. MMC-induced conditions for each O113:H21 strain. A one-way ANOVA with Tukey’s multiple comparisons test was used to compare ST groups for both non-induced and MMC-induced conditions. Statistical significance for *N*=2 experiments is reported as *P < 0.05; **P < 0.005; ***P < 0.0005; ****P < 0.00005. The Stx levels produced under non-induced conditions by strains from both clonal groups were indiscriminate; however, Stx2 litres recorded under MMC-induced conditions were found to be elevated at statistically significant levels in ST-820 complex isolates, suggesting increased pathogenicity.

Indeed, the most potent cytopathic toxin subtype Stx_2a_ is commonly found in the O113:H21 serotype [55]. Using Stx_2_-ELISA, we assessed toxin production under both spontaneous and Stx-phage mobilizing conditions (Fig. 9), which allowed for cross-comparison of ST-820 and ST-223 Stx_2a_-production pathotypes (Table S1). We recorded toxin production under both non-induced and Stx-phage mobilizing conditions (Fig. 9). Induction efficiency of the Stx-phages is positively correlated to Stx-production [161–166], and widely used as a means to assess an STEC strains’ individual Stx-conferred pathogenic potential (Fig. 9) [24, 25, 28, 35, 39, 153, 167–172]. As expected, the Stx_2_-cytotoxicity in ST-820 and ST-223 cultures was exacerbated by MMC-treatment, which triggers the RecA-mediated SOS response by causing DNA damage that leads to lytic Stx_2_ phage activation and ultimately toxin production [3] (Fig. 9). The amounts of Stx in the biological replicates were reproducible and showed a strong correlation to the known strain-associated epidemiological metadata. The Stx-levels produced under non-induced conditions were indiscriminate between ST-820 and ST-223 strains, however, Stx2 litres recorded under MMC-induction were found to be elevated at statistically significant levels in ST-820 complex isolates, and thus may increase pathogenicity. Strain EH53 is an outlier amongst ST-820 strains showing low Stx-production under both induced and non-induced conditions (Fig. 9). The integrity of the Stx_2a_-phage seems unaltered, though its fragmented status on three contigs prevents a more detailed analysis (data not shown) (Table S3). Within ST-223, we identified strains MDP9-27, 10, and TW02918, the latter isolated from a patient with diarrhoea, with significantly increased Stx-levels comparable to those recorded for ST-820 (Fig. 9). Strains with these genotypes could be potentially pathogenic to humans, which raises concerns should such isolates enter the human food chain. The actual disease outcome, however, cannot be predicted. Stx-production is a definite factor in causing disease, though disease severity is a result of complex interactions between STEC, the host microbiota [8, 50, 52, 173–178], and the age and immunogenetics of the infected patient, with higher incidence of HUS typically in the young and elderly [179–181]. All these factors may independently or in combination impact Stx-production and ultimately the outcomes of severe pathogenesis in humans [182].

## Discussion

This study serves as basis to identify genomic signatures associated with hypervirulent Stx-producing, HUS-causing subpopulations in STEC O113:H21. Integrating genomic and virulence information following genome-wide association studies (GWAS) principles [183–185] opens the avenue for improved typing, biosurveillance and risk assessment and may allow the identification of circulating hypervirulent STEC subpopulations [19, 32, 42, 186]. Insights into the pathogenome make-up and associated virulence traits is foundational to understand the evolutionary trajectories of STEC lineages [187–189], which in the case of O113:H21, resulted in the phylogeographical diversification in the ST-820 and ST-223 complexes. The established phylogenomic framework allowed us to refine the genome and virulence boundaries between these two major complexes and identified a quasi-intermediate position for the historic non-shigatoxigenic ST-223 strain 6182–50 from Mexico. Since plasmids may get lost during laboratory cultivation or often recovered only in fragments, typing efforts are mostly focused on stable chromosomal markers. However, our data suggest that plasmid information may be used to further refine the phylogenetic model. Plasmid comparison revealed a correlation between the strains’ pO113 plasmid genotype and chromosomally inferred sequence type, which suggests the coevolution of the chromosome and accessory plasmids. In accordance with the epidemiological metadata, we further describe significant differences in Stx_2a_-production capabilities of ST-223 and ST-820 complex strains. The latter may possess an increased pathogenic potential (Fig. 9). This is particularly evident under MMC-induction, a condition that mimics the Stx-phage induction [190–195]. In this context, we note that the definition of pathogenic potential is often skewed by anthropogenic biases, such as fitness factors that are not, per se, accounted for in the virulence inventory and may allow pathogens to access the production foods for human consumption [196, 197]. Our lineage-scale study did not identify a distinct virulence gene profile associated with either ST. However, the noted complex-specific trends in the *in vitro* Stx_2a_-production pathotypes may suggest alterations in the underlying dynamic regulatory networks [198–200], which is the focus of our ongoing research. The O113:H21 lineage is not one of the so called ‘Big Six’ non-O157 serogroups of high-risk concern [201], and at present, O113:H21 strains have not caused major outbreaks in the US. Still, some of the tested ST-223 strains from contaminated produce or bovine reservoir, showed Stx-production levels comparable to ST-820 isolates and are likely capable of causing disease in humans. Taken together, our findings call for increased awareness and continued surveillance of this serotype. Clearly, further research is required to elucidate how the individual O113:H21 genotype relates to human disease by taking into account pathogenicity traits that were not examined, such as biofilm formation, adherence and invasiveness [66, 198, 199, 202–206], production of non-Stx toxins [207, 208] and other putative STEC virulence factors.

## Supplementary Data

Supplementary material 1

Supplementary material 2
