# Supplementary material for: Pathogenomes and variations in Shiga toxin production among geographically distinct clones of Escherichia coli O113:H21
Source: Microb Genom. 2022 Apr 8;8(4):000796. doi: 10.1099/mgen.0.000796 (PMC9453080; doi:10.1099/mgen.0.000796)
Supplement: Supplementary material 1 [file mgen-8-0796-s001.pdf]

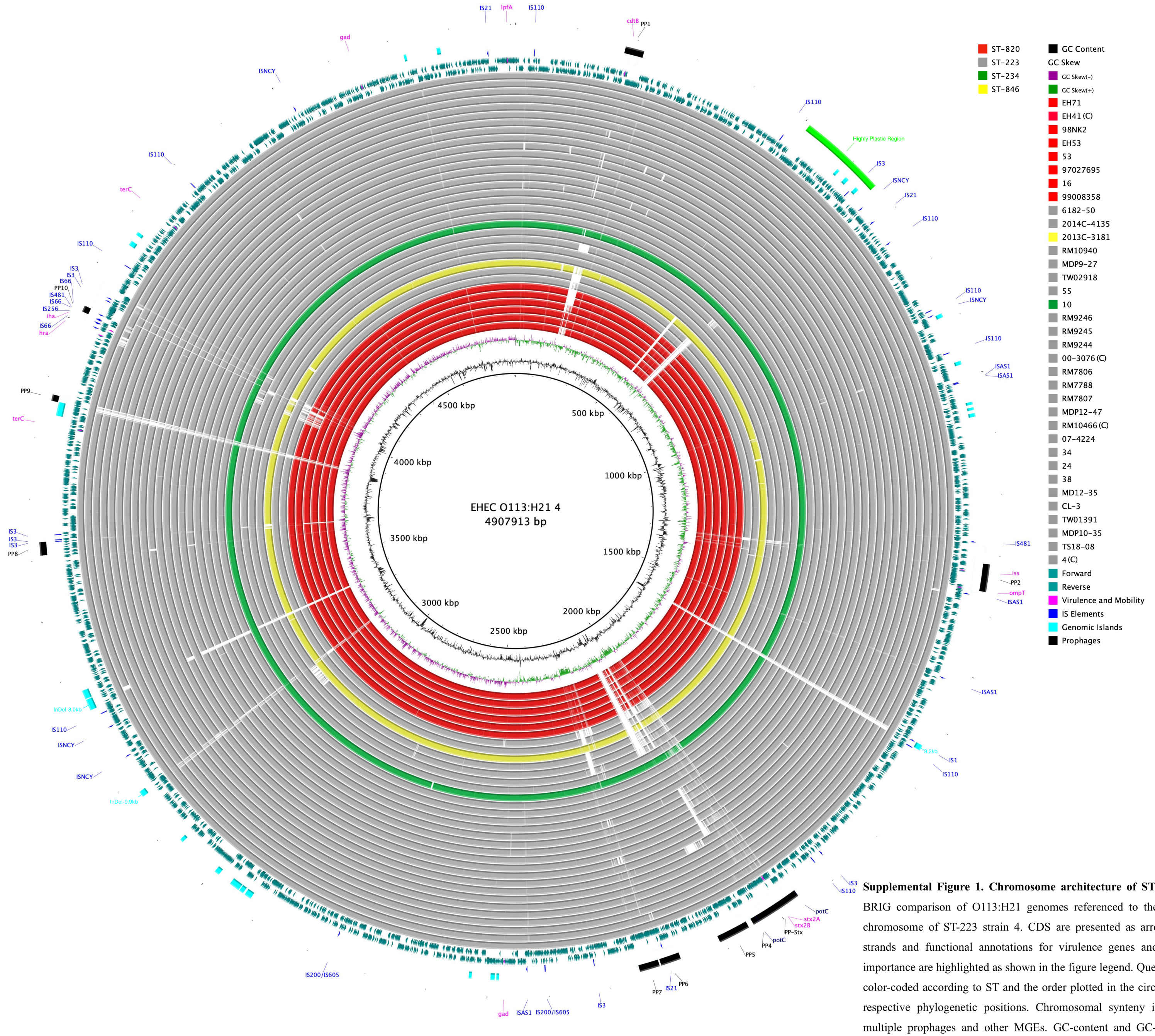

Supplemental Figure 1. Chromosome architecture of STEC O113:H21

BRIG comparison of O113:H21 genomes referenced to the 4,907,913 bp chromosome of ST-223 strain 4. CDS are presented as arrows on the +/- strands and functional annotations for virulence genes and other loci of importance are highlighted as shown in the figure legend. Query genomes are color-coded according to ST and the order plotted in the circle reflects their respective phylogenetic positions. Chromosomal synteny is disrupted by multiple prophages and other MGEs. GC-content and GC-skew of the 4 chromosome are depicted in the two innermost circles. (C) denotes closed chromosomes.

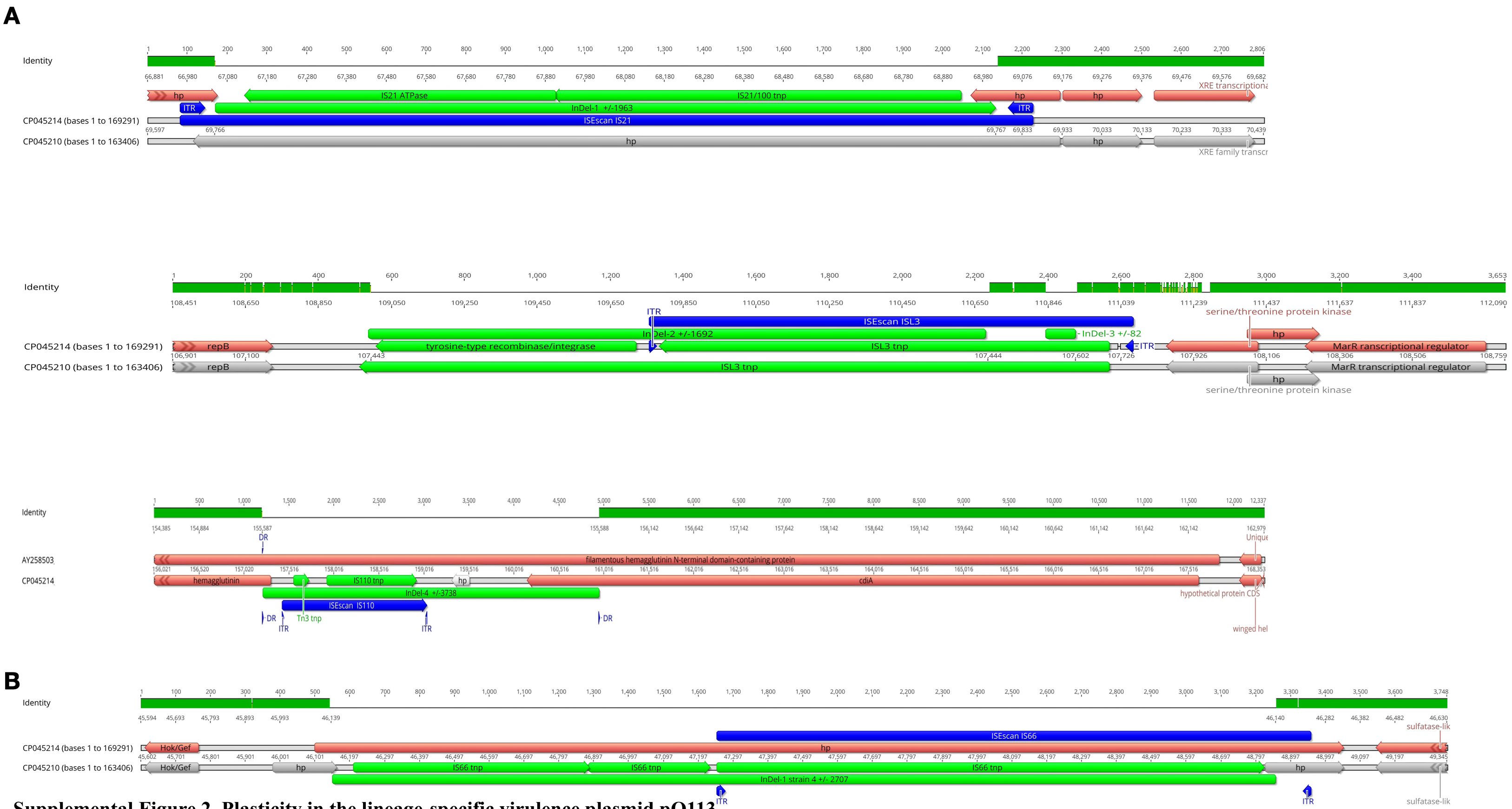

Supplemental Figure 2. Plasticity in the lineage-specific virulence plasmid pO113

(A) referenced to the closed pO113-EH41 (ST-820) and **(B)** pO113-4 (ST-223) plasmids.

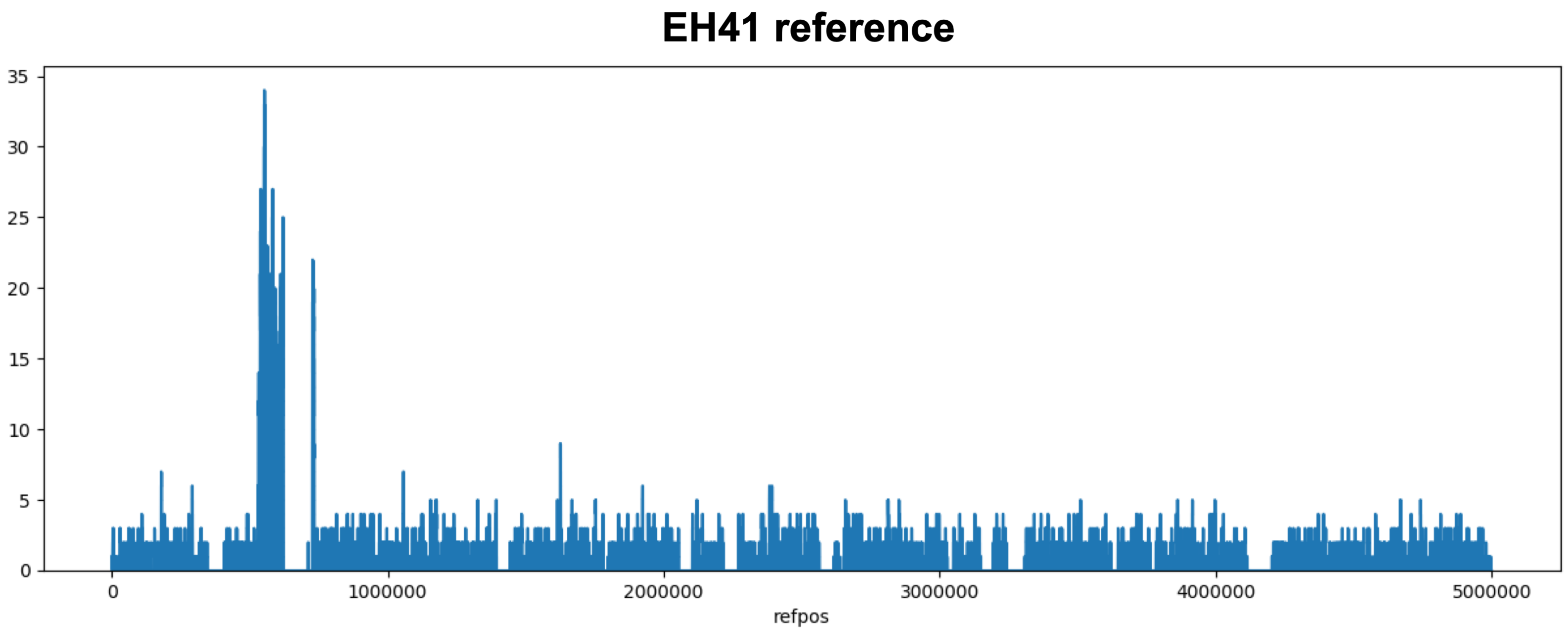

Supplemental Figure 3. Chromosome-wide distribution of SNPs

The positions of the 4,398 identified SNPs were plotted along the EH41 chromosome using a 1,000 bp sliding window. Deserted regions lacking any SNP calls correspond to excluded MGEs, such as prophages or islands, and repeats. SNPs were found dispersed throughout the chromosome; however, we detected elevated SNP density localized in a region of high plasticity (see also **Fig. 1**). Of note, SNPs in this region separate ST-223 from ST-820 complex isolates, suggesting a potential role for this region in the phylogeographical diversification of O113:H21.

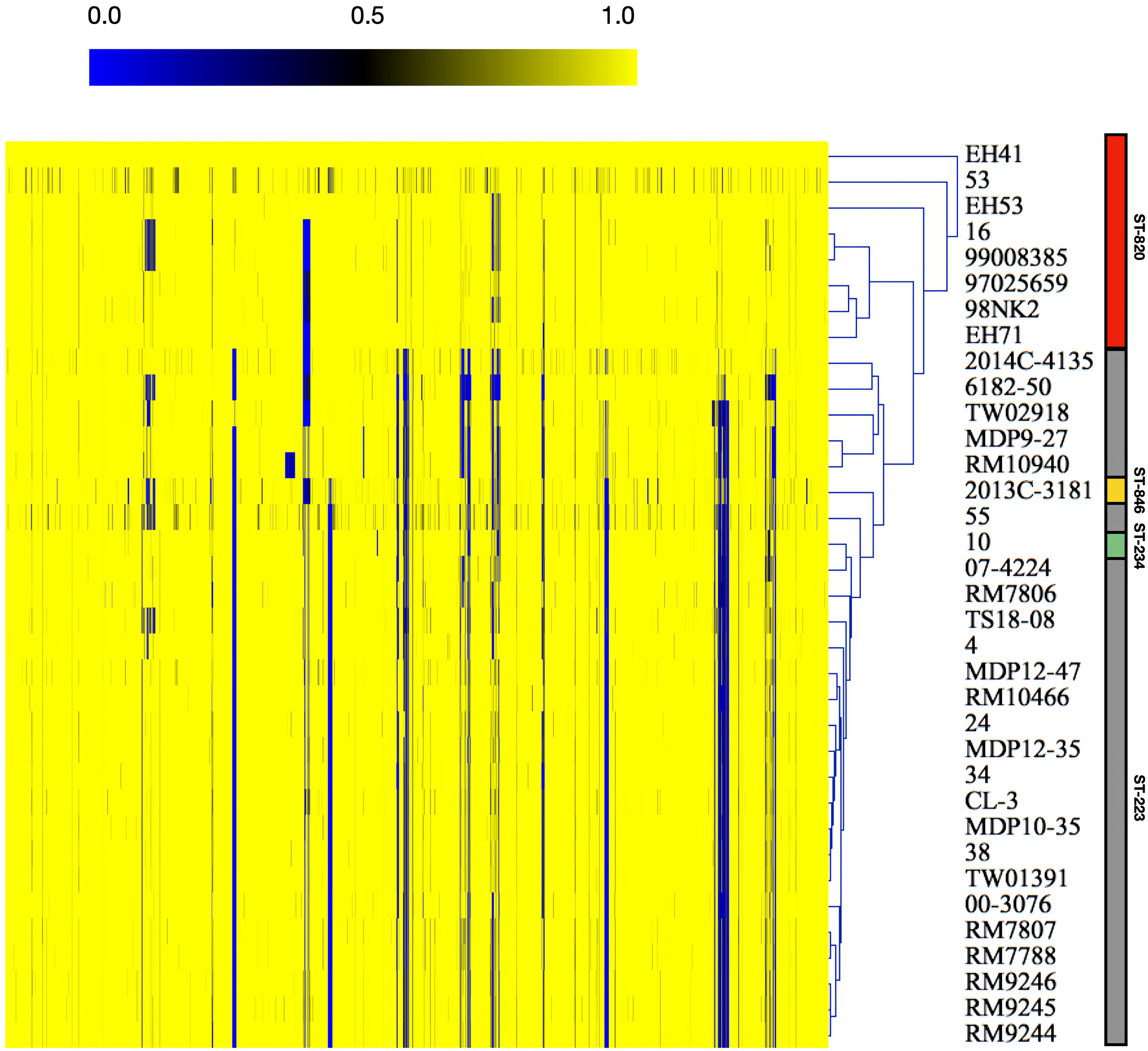

Supplemental Figure 4. Hierarchical cluster heatmap of proteomes

The proteomes of 35 O113:H21 strains were compared by LS-BSR [138] and referenced to the proteome of closed ST-820 strain EH41. The resulting BSR scores were converted into a distance matrix and used in MeV v4.8 [140] to construct a heatmap showing the prevalence of protein coding genes with values ranging from 0 (blue, absent) to 1 (yellow, identical). Hierarchical average linkage clustering by Pearson correlation separated ST-820 strains from ST-223 strain, a finding in accordance with the established wgMLST-, WGA- and SNP-derived phylogenies. The heatmap also confirms the quasi-intermediate position of ST-223 strains 2014C-4135 and 6182-50 and further, the relatively distant position of ST-846 strain 2013C-3181, which is separated by 1774 allele changes from the ST-223 complex.
